# Quantum Nondemolition Measurement of a Nonclassical State of a Massive Object

**DOI:** 10.1103/PhysRevX.5.041037

**Published:** 2015-12-07

**Authors:** F. Lecocq, J. B. Clark, R.W. Simmonds, J. Aumentado, J. D. Teufel

**Affiliations:** National Institute of Standards and Technology, 325 Broadway, Boulder, Colorado 80305, USA

**Keywords:** Quantum Physics, Quantum Information

## Abstract

By coupling a macroscopic mechanical oscillator to two microwave cavities, we simultaneously prepare and monitor a nonclassical steady state of mechanical motion. In each cavity, correlated radiation pressure forces induced by two coherent drives engineer the coupling between the quadratures of light and motion. We, first, demonstrate the ability to perform a continuous quantum nondemolition measurement of a single mechanical quadrature at a rate that exceeds the mechanical decoherence rate, while avoiding measurement backaction by more than 13 dB. Second, we apply this measurement technique to independently verify the preparation of a squeezed state in the mechanical oscillator, resolving quadrature fluctuations 20% below the quantum noise.

## I. INTRODUCTION

While quantum mechanics exquisitely describes the behavior of microscopic systems, one ongoing challenge is to explore its applicability to systems of larger size and mass. Unfortunately, quantum states of increasingly macroscopic objects are more easily corrupted by unintentional measurements from the classical environment. Additionally, even the intentional measurements from the observer can further perturb the system [1]. In optomechanics [2], coherent light fields serve as the intermediary between the fragile mechanical states and our inherently classical world by exerting radiation pressure forces and extracting mechanical information. Here, we engineer a microwave cavity optomechanical system [3] to stabilize a nonclassical steady state of motion while independently, continuously, and nondestructively monitoring it. By coupling the motion of an aluminum membrane to two microwave cavities, we separately prepare a squeezed state of motion [4] and monitor it with a quantum nondemolition (QND) measurement [5–7]. We resolve subvacuum mechanical quadrature fluctuations, characteristic of a state that has no classical analog [8]. The techniques developed here have direct applications [9] in the areas of quantum-enhanced sensing [10] and quantum information processing, and could be further extended to more complex quantum states [11].

Reflecting light off a mechanical object induces a momentum transfer, allowing one to control and measure the mechanical state. When the photon scattering rate exceeds the phonon decoherence rate, the mechanical system becomes more strongly coupled to the photon reservoir than to its own thermal environment. This regime is usually obtained by embedding a mechanical resonator into an electromagnetic cavity to increase the interaction strength per photon [2]. Additionally, the cavity filters the density of states available for the scattered photons, allowing control over the ratio of Stokes and anti-Stokes scattering rates. Importantly, the nature of the optomechanical interaction implies that the light field interacts with both mechanical quadratures, with fundamental consequences on the mechanical state preparation and measurement. On one hand, the precision on the simultaneous measurement of both mechanical quadratures is limited by the Heisenberg uncertainty principle [12,13]. On the other hand, the state preparation via sideband cooling exploits the coherent exchange of the cavity and mechanical state and is therefore limited by the statistics of the classical light field [14–16].

Both limitations can be overcome using polychromatic coherent light. One can address and manipulate each mechanical quadrature differently by engineering interference processes between their couplings to the cavity quadratures. More specifically, a two-drive scheme can be used to design a single quadrature measurement of the mechanical oscillator, known as backaction evading [5–7]. The scheme fulfills the requirement for a QND measurement, which is an important tool for the tomographic reconstruction of arbitrary quantum states. A similar scheme was proposed by Kronwald *et al.* [4] to prepare a mechanical squeezed state, following an analogous idea formulated for trapped ions [17,18]. It was very recently implemented in optomechanical systems [19,20]; however, the mechanical squeezing was not independently measured, but only inferred from the output spectrum of the squeezing operation itself. In this work, we separate the state preparation and its read-out by coupling a macroscopic mechanical oscillator to two microwave cavities. We use an independent QND measurement to perform the tomography of a squeezed state of a macroscopic mechanical oscillator.

## II. THEORY

Consider a cavity optomechanical system where the position of a mechanical resonator of frequency Ω*_m_* tunes the resonance frequency *ω_c_* of an electromagnetic cavity [2]. Two drives are applied to the cavity, at both mechanical sidebands *ω*^±^ = *ω_c_* ± Ω*_m_*. The strength of each drive can be parametrized by its scattering rate $\Gamma^{\pm }=4g_{0}^{2}n^{\pm }/\kappa$, where *g*_0_ is the vacuum optomechanical coupling rate, *κ* is the cavity linewidth, and *n*^±^ is the number of intracavity photons induced by each drive. Assuming a mechanical relaxation rate Γ*_m_* and the condition Γ*_m_*, Γ^±^ ≪ *κ* ≪ Ω*_m_*, one can write the relations between the mechanical quadrature amplitudes, *X̂*_1_ and *X̂*_2_, and the amplitude and phase quadrature of the cavity fields, *Â* and *φ̂*, reading [4]:

(1) $$\langle {\hat{X}}_{1}^{2}\rangle =\frac{\Gamma_{m}\langle {\hat{X}}_{\text{th}}^{2}\rangle +{\left(\sqrt{\Gamma^{-}}-\sqrt{\Gamma^{+}}\right)}^{2}\langle {\hat{\varphi }}^{2}\rangle }{\Gamma_{m}+\Gamma^{-}-\Gamma^{+}},$$

(2) $$\langle {\hat{X}}_{2}^{2}\rangle =\frac{\Gamma_{m}\langle {\hat{X}}_{\text{th}}^{2}\rangle +{\left(\sqrt{\Gamma^{-}}+\sqrt{\Gamma^{+}}\right)}^{2}\langle {\hat{A}}^{2}\rangle }{\Gamma_{m}+\Gamma^{-}-\Gamma^{+}}.$$

Here, $\langle X_{\text{th}}^{2}\rangle =2n_{m}^{\text{th}}+1$ is the variance of the mechanical quadratures for an equilibrium thermal occupancy $n_{m}^{\text{th}}$ and 〈*φ̂*^2^〉 = 〈*Â*^2^〉 = 1 are the variances of the cavity quadratures for an ideal coherent state. For Γ^+^ = 0, corresponding to driving only the lower sideband, one recovers the sideband cooling limit, and at high scattering rate, Γ^−^ ≫ Γ*_m_*, each quadrature of the mechanics is cooled to the cavity quadratures, $\langle {\hat{X}}_{1}^{2}\rangle =\langle {\hat{\varphi }}^{2}\rangle$ and $\langle {\hat{X}}_{2}^{2}\rangle =\langle {\hat{A}}^{2}\rangle$. Another limit is Γ^+^ = Γ^−^, corresponding to driving symmetrically the upper and lower sidebands. This is the case of a drive on resonance with the cavity whose amplitude is modulated at a mechanical frequency, performing a QND measurement of the mechanical quadrature *X̂*_1_. Indeed, under these conditions, Eqs. (1) and (2) read $\langle {\hat{X}}_{1}^{2}\rangle =\langle {\hat{X}}_{\text{th}}^{2}\rangle$ and $\langle {\hat{X}}_{2}^{2}\rangle =\langle {\hat{X}}_{\text{th}}^{2}\rangle +(4\Gamma^{-}/\Gamma_{m})\langle {\hat{A}}^{2}\rangle$. The *X̂*_1_ quadrature is unaffected by the measurement, and the backaction from radiation pressure shot noise is placed on the orthogonal quadrature *X̂*_2_. Finally, the preparation of a squeezed state occurs in the intermediate regime, Γ^+^ < Γ^−^. The mechanical mode is coupled, at a reduced rate Γ^−^ − Γ^+^, to an effectively squeezed microwave bath, whose minimum variance is ${\left(\sqrt{\Gamma^{-}}-\sqrt{\Gamma^{+}}\right)}^{2}/(\Gamma^{-}-\Gamma^{+})<1$.

## III. RESULTS

**FIG. 1:**
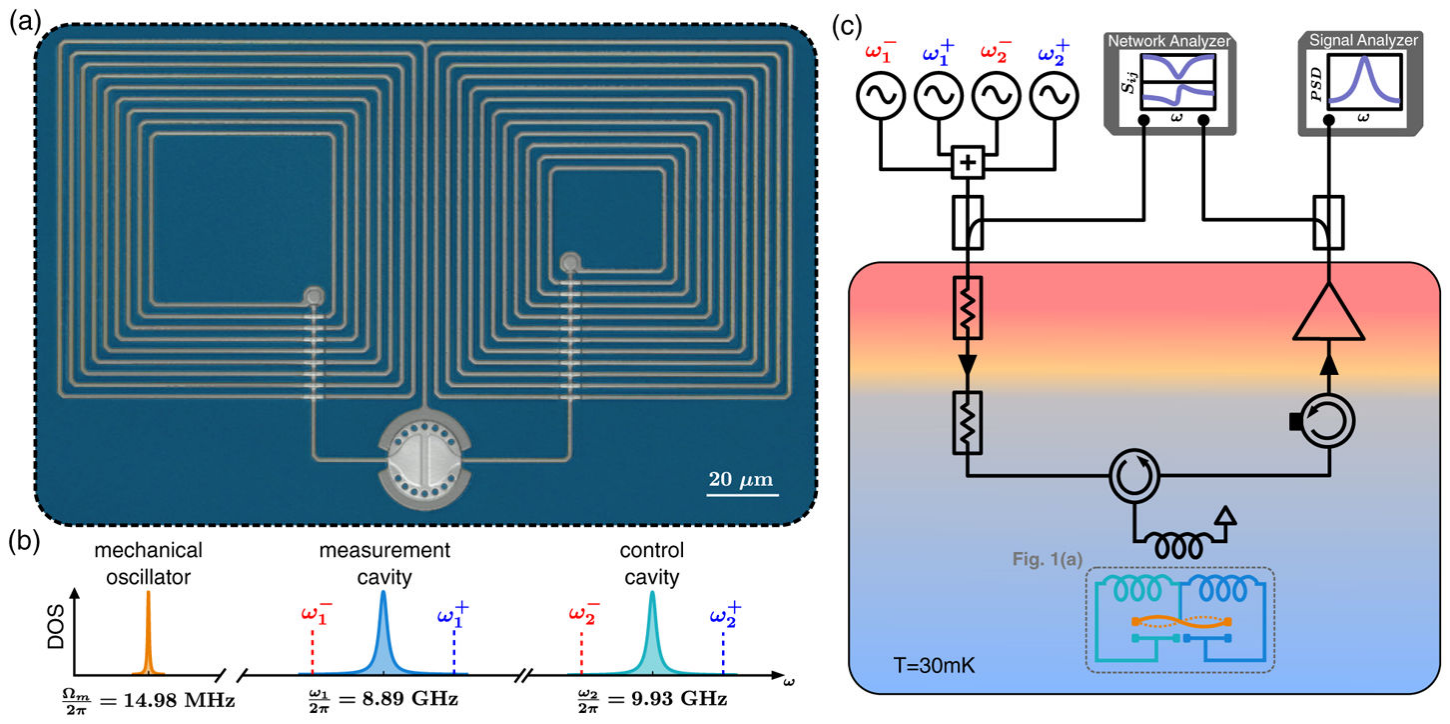
Device description and experimental setup. (a) False-color optical micrograph of the aluminum device (in gray) on a sapphire substrate (blue). Centered at the bottom of the micrograph is a mechanically compliant vacuum gap capacitor. The capacitor’s electrode is split into two plates, each shunted by a different coil inductor, giving rise to two microwave resonances. (b) Frequency space diagram. Except for the mechanical linewidth, all the frequencies and linewidths are to scale. The microwave cavities have Lorentzian densities of states (DOS) of width *κ*_1_/2*π* = 1.7 MHz and *κ*_2_/2*π* = 2.1 MHz, centered at *ω*_1_/2*π* = 8.89 GHz and *ω*_2_/2*π* = 9.93 GHz. These resonance frequencies are tuned by the motion of the top plate of the capacitor, at the mechanical frequency Ω*_m_*/2*π* = 14.98 MHz. The red and blue dashed lines indicate the four mechanical sideband frequencies at which the cavities are driven, $\omega_{1,2}^{\pm }=\omega_{1,2}\pm \Omega_{m}$. (c) The circuit is placed on the cold stage of a cryogenic refrigerator (base temperature *T* = 30 mK). Up to four strong microwave drives and one weak microwave probe are inductively coupled to the cavities via a single port. The reflected signals and the noise emitted by the cavities are amplified at low temperature and demodulated at room temperature.

In order to separately prepare and read-out a mechanical state, we engineer a microwave optomechanical system where a single mechanical mode is coupled to two microwave cavities. The experimental setup is shown in Fig. 1. The circuit, made out of aluminum on a sapphire substrate, consists of a central vacuum gap capacitor shunted by two coil inductors [3,21]. The bottom plate of the capacitor is split to create two cavity resonances, *ω*_1_/2*π* = 8.89 GHz and *ω*_2_/2*π* = 9.93 GHz, named, respectively, the “measurement cavity” and the “control cavity.” The top plate of the capacitor is mechanically compliant, with a second harmonic mode of motion resonating at Ω*_m_*/2*π* = 14.98 MHz (see Appendix A). Its motion tunes the resonance of both microwave cavities, with respective vacuum optomechanical couplings *g*_1_/2*π* = 145 Hz and *g*_2_/2*π* = −170 Hz (see Appendix B). Operated at a temperature of *T* = 30 mK, the equilibrium mechanical thermal occupancy is $n_{m}^{\text{th}}=42$ phonons and the mechanical relaxation rate is Γ*_m_*/2*π* = 9.2 Hz. Both microwave cavities are strongly overcoupled to a single measurement port, setting their linewidths to *κ*_1_/2*π* = 1.7 MHz and *κ*_2_/2*π* = 2.1 MHz. This coupling ensures that internal dissipations contribute by less than 5% to the total linewidths, while maintaining a strongly resolved sideband regime, Ω*_m_*/*κ*_1,2_ > 7. It also thermalizes the cavities to the shot-noise-limited input fields, maintaining throughout this work a thermal cavity occupancy well under our measurement noise floor, $n_{c}^{\text{th}}<0.1$.

**FIG. 2:**
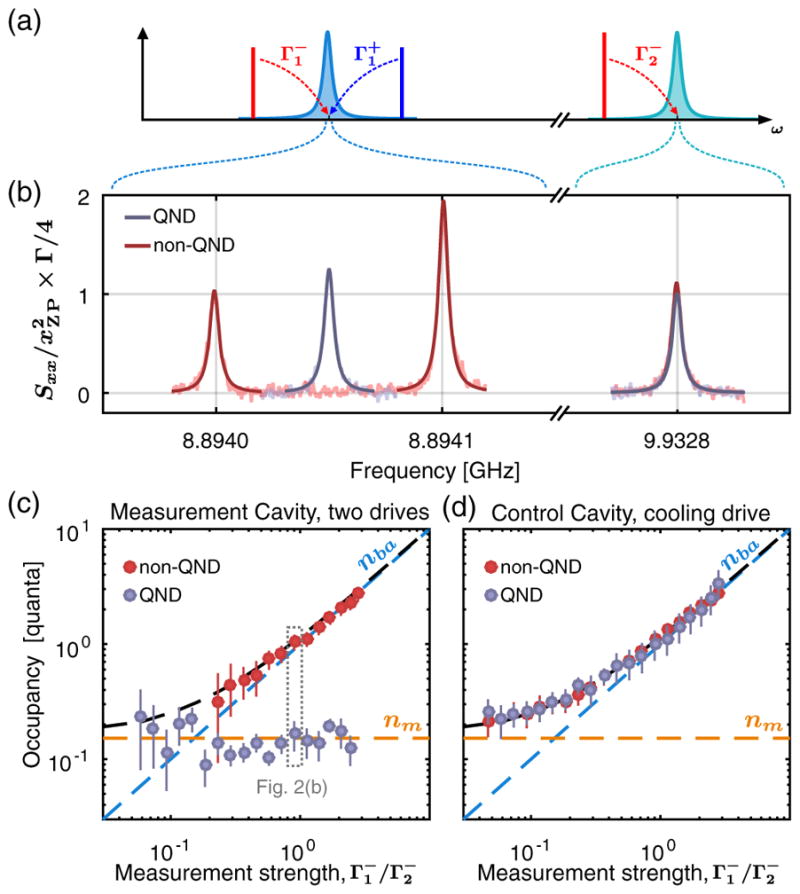
Quantum nondemolition measurement. (a) Measurement schematic. A cooling drive of strength $\Gamma_{2}^{-}=2\pi \times 4.87\;\text{kHz}=529\Gamma_{m}$ is applied on the lower mechanical sideband of the control cavity ( $\omega_{2}^{-}=\omega_{2}-\Omega_{m}$). Two drives of equal strength $\Gamma_{1}^{-}=\Gamma_{1}^{+}$ are applied close to the mechanical sidebands of the measurement cavity. Their frequencies can be tuned to $\omega_{1}^{\pm }=\omega_{1}\pm \Omega_{m}$ to perform a single mechanical quadrature measurement [QND, in gray in (b)–(d)] or detuned by many mechanical linewidths away from that optimum to measure both mechanical quadratures [non-QND, in red in (b)–(d)]. (b) Mechanical noise spectra (normalized, background subtracted), for $\Gamma_{1}^{-}/\Gamma_{2}^{-}=0.9$. (c),(d) Mechanical occupancy extracted from the measured spectra of the two-drives measurement (c) and cooling drive (d), for both the non-QND case (in red) and the QND case (in gray), as a function of the measurement strength $\Gamma_{1}^{-}/\Gamma_{2}^{-}$.

We start by describing the QND measurement of the mechanical oscillator, cooled close to its ground state, in Fig. 2. A cooling drive of strength $\Gamma_{2}^{-}=2\pi \times 4.87\;\text{kHz}=529\Gamma_{m}$ is applied at the lower mechanical sideband of the control cavity, $\omega_{2}^{-}=\omega_{2}-\Omega_{m}$, leading to a reduced mechanical thermal occupancy *n_m_*. Simultaneously, two drives of equal strength, $\Gamma_{1}^{-}=\Gamma_{1}^{+}$, are applied close to the mechanical sidebands of the measurement cavity, acting back on the mechanical oscillator [7] and increasing the total occupancy to $n_{m}^{\text{tot}}=n_{m}+n_{ba}$, where $n_{ba}=\Gamma_{1}^{-}/\Gamma_{2}^{-}$. Their frequencies can be optimally tuned to $\omega_{1}^{\pm }=\omega_{1}\pm \Omega_{m}$ to perform a single mechanical quadrature measurement [QND, in gray in Figs. 2(b)–2(d)] or detuned by many mechanical linewidths away from that optimum to measure both mechanical quadratures [non-QND, in red in Figs. 2(b)–2(d)]. By monitoring the driven responses of both cavities [3,22,23], we tune very precisely the strength of each drive and measure all the mode frequencies and decay rates (see Appendix C). We then acquire the noise power emitted by both cavities to extract the mechanical state (see Appendix D). In Fig. 2(b), we fix the measurement rate to $\Gamma_{1}^{-}=\Gamma_{1}^{+}=0.9\Gamma_{2}^{-}$, and show the measured spectra, normalized to mechanical units.

In the non-QND case, each drive measures both mechanical quadratures, and the noise power of each thermomechanical sideband is proportional to $n_{m}^{\text{tot}}$ or $n_{m}^{\text{tot}}+1$ for the anti-Stokes and Stokes scattering, respectively [24]. Note that this sideband asymmetry [25,26] provides a primary calibration of the *y* axis, in good agreement with the independently measured coupling strengths *g*_1_ and *g*_2_. From each sideband, we extract the same mechanical occupancy $n_{m}^{\text{tot}}$, shown as a function of measurement strength in Figs. 2(c) and 2(d). The measured quantum backaction scales ideally with the measurement strength, and we can extrapolate a mechanical thermal occupancy of *n_m_* = 0.15 ± 0.05.

We now tune the frequency of the measurement drives to the QND case. As shown in Fig. 2(b), the noise sideband of the cooling drive is unchanged. Indeed, that drive still measures both mechanical quadratures, accessing the same total mechanical occupancy as in the non-QND measurement [see Fig. 2(d)]. On the contrary, on the measurement cavity, the mechanical sidebands of each drive interfere with each other when brought into the QND case, leaving a single Lorentzian noise peak proportional to the variance of a single mechanical quadrature given by $\langle {\hat{X}}_{1}^{2}\rangle =2n_{m}+1$. The backaction has been evaded and placed on the orthogonal quadrature, conserving the total mechanical occupancy [see Eqs. (1) and (2)]. As expected, the measured mechanical thermal occupancy is constant as a function of the measurement strength, and quantitatively agrees with the occupancy inferred in the non-QND case. At the measurement strength of $\Gamma_{1}^{-}/\Gamma_{2}^{-}=2.44$, we can place a conservative upper bound on the residual quantum measurement backaction, at about 0.1 quanta, corresponding to a reduction of the backaction by more than 13 dB. This demonstrates a QND measurement of a single mechanical quadrature at a rate much faster than the mechanical decoherence rate.

**FIG. 3:**
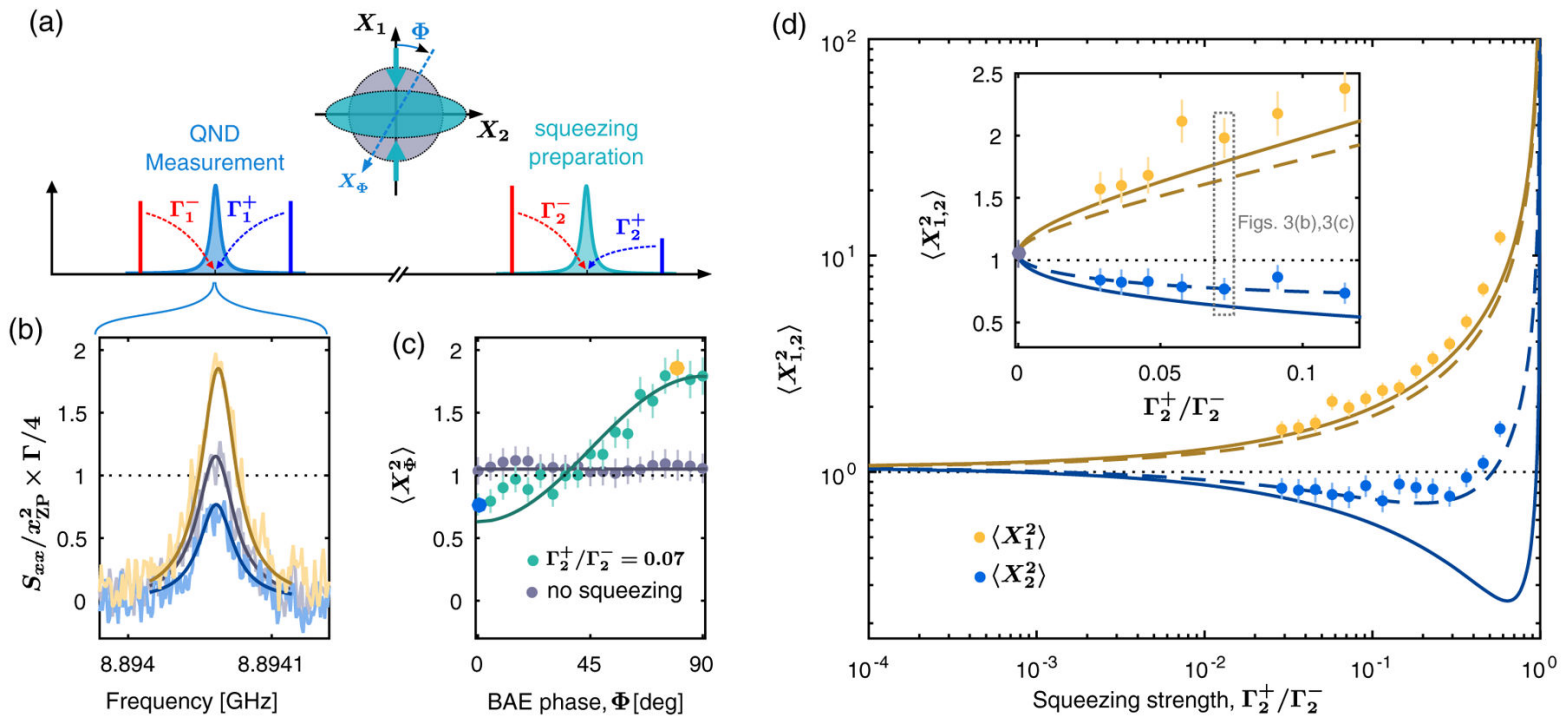
Tomography of a mechanical squeezed state. (a) Measurement schematic. A pair of drives at $\omega_{2}^{\pm }=\omega_{2}\pm \Omega_{m}$ cools the mechanical mode to a squeezed bath, while a pair of drives at $\omega_{1}^{\pm }=\omega_{1}\pm \Omega_{m}$ measures the generalized mechanical quadrature *X̂*_Φ_, given by the tunable phase Φ, allowing for the tomographic measurement of the squeezed state. (b) Normalized noise spectra of the QND measurement (background subtracted), for the squeezed and antisqueezed quadratures ( $\Gamma_{2}^{-}/\Gamma_{2}^{+}=0.07$) in blue and yellow, respectively, compared to a spectrum measured without squeezing ( $\Gamma_{2}^{+}=0$) in gray. (c) Measured quadrature variances as a function of phase for $\Gamma_{2}^{+}=0$ in gray and $\Gamma_{2}^{-}/\Gamma_{2}^{+}=0.07$ in green. The spectra in (b) correspond to the blue and yellow dots. (d) Squeezed and antisqueezed quadrature variances, respectively, in blue and yellow, as a function of the squeezing strength $\Gamma_{2}^{-}/\Gamma_{2}^{+}$. The solid lines are the theoretical predictions with no free parameters. The dashed lines are the theoretical predictions including a drift of the measurement phase of 35 deg during the acquisition of each spectrum (see Appendix E). The inset is a zoom-in for the low squeezing strength data, on a linear scale, where we can place the variance measured without squeezing (gray dot). In all figures, the black dotted line is the vacuum limit.

This measurement scheme allows us to perform the tomography of the mechanical state, described in Fig. 3. Indeed, we can control the generalized mechanical quadrature being measured, $\hat{X_{\Phi }}$, by simply rotating the relative phase between the measurement drives. As we expect the state to be Gaussian, the measurement of the second moment of the noise is sufficient to reconstruct its tomogram. A mechanical state prepared by simple sideband cooling is expected to have equal variances for each quadrature. In Fig. 3(c), we show data for an increased cooling strength $\Gamma_{2}^{-}=2\pi \times 15.11\;\text{kHz}=1643\Gamma_{m}$ and a measurement strength $\Gamma_{1}^{-}/\Gamma_{2}^{-}=0.48$. As expected, the results of the QND measurements are phase independent. We measure a mechanical occupancy *n_m_* < 0.1, demonstrating the QND measurement of a highly pure Gaussian state.

We now apply this same tomographic measurement to verify the preparation of a nonclassical state of motion. We prepare a squeezed state by adding a drive of strength $\Gamma_{2}^{+}$ at the upper mechanical sideband of the control cavity ( $\omega_{2}^{+}=\omega_{2}+\Omega_{m}$). Again, as a squeezed state is still Gaussian, we simply measure the variance of the mechanical quadrature $\hat{X_{\Phi }}$ as a function of the measurement phase to fully characterize the state, as shown in Fig. 3(c) for a squeezing strength $\Gamma_{2}^{+}/\Gamma_{2}^{-}=0.07$. We resolve a minimum quadrature variance below vacuum, $\langle {\hat{X}}_{1}^{2}\rangle =0.78\pm 0.08$. The spectra corresponding to the measurement of the squeezed and antisqueezed quadratures are shown in Fig. 3(b). Finally, in Fig. 3(d), we plot the variance of the squeezed and antisqueezed quadratures, 〈 ${\hat{X}}_{1}^{2}$〉 and 〈 ${\hat{X}}_{2}^{2}$〉, respectively, as a function of the squeezing strength. The solid lines in Figs. 3(c) and 3(d) are theoretical predictions from Eqs. (1) and (2) without free parameters, in reasonable agreement with the data at low squeezing strength. As the strength of the squeezing is increased, our data begin to deviate significantly from the ideal theory. While many noise mechanisms could, in principle, degrade the measured squeezing, one plausible candidate is the variation of the relative phase between the squeezing operation and the quadrature measurement during the averaging. A model assuming a phase variation of 35 deg reproduces the qualitative behavior of the observed squeezing (dashed lines). While our independent measurement of the phase stability (see Appendix E) does not fully account for the variation required to fit the data, it does suggest that future experiments could benefit from improved phase stabilization between the four pumps and the two cavity modes.

Looking forward, the introduction of stronger nonlinearities, combined with reservoir engineering, would enable the preparation of more complex quantum states [11], further motivating the use of mechanical systems as ultrasensitive detectors and quantum memories [9]. We emphasize that acquiring all the moments of the noise emitted by the QND measurement would allow us to reconstruct an arbitrary quantum state [27,28]. Additionally, the inherent nonlinearity of optomechanical cavities can act as a nearly ideal mixing element, opening routes for innovative types of amplification [29], frequency conversion [30], and nonreciprocal behavior [31].

## Figures and Tables

**FIG. 4:**
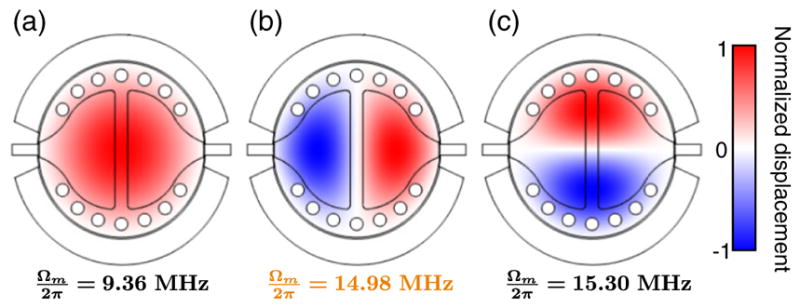
Mechanical mode shape. (a)–(c) Finite element simulation of the out-of-plane displacement of the first three mechanical modes of the aluminum membrane, and their respective measured resonance frequencies. The mode used in the main text is shown in (b).

**FIG. 5:**
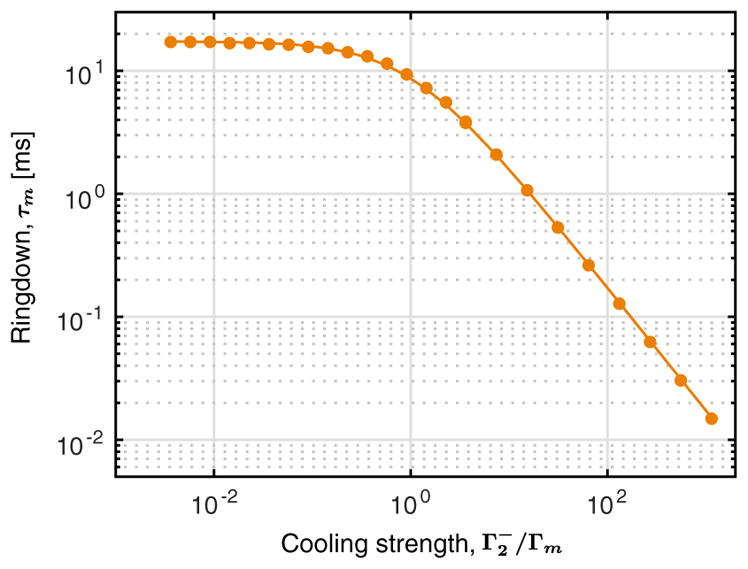
Mechanical ringdown time. We measure the mechanical ringdown time *τ_m_* as a function of the strength of a cooling drive on the control cavity, at a frequency $\omega_{2}^{-}=\omega_{2}-\Omega_{m}$, scattering light and damping the mechanical oscillator at the rate $\Gamma_{2}^{-}$. The solid line is the theoretical prediction from the total damping, $\Gamma_{\text{tot}}=\Gamma_{m}+\Gamma_{2}^{-}$. We measure an intrinsic mechanical ringdown time of *τ_m_* = 17.3 ms, corresponding to a relaxation rate Γ*_m_* = 1/*τ_m_* = 2*π* × 9.2 Hz.

**FIG. 6:**
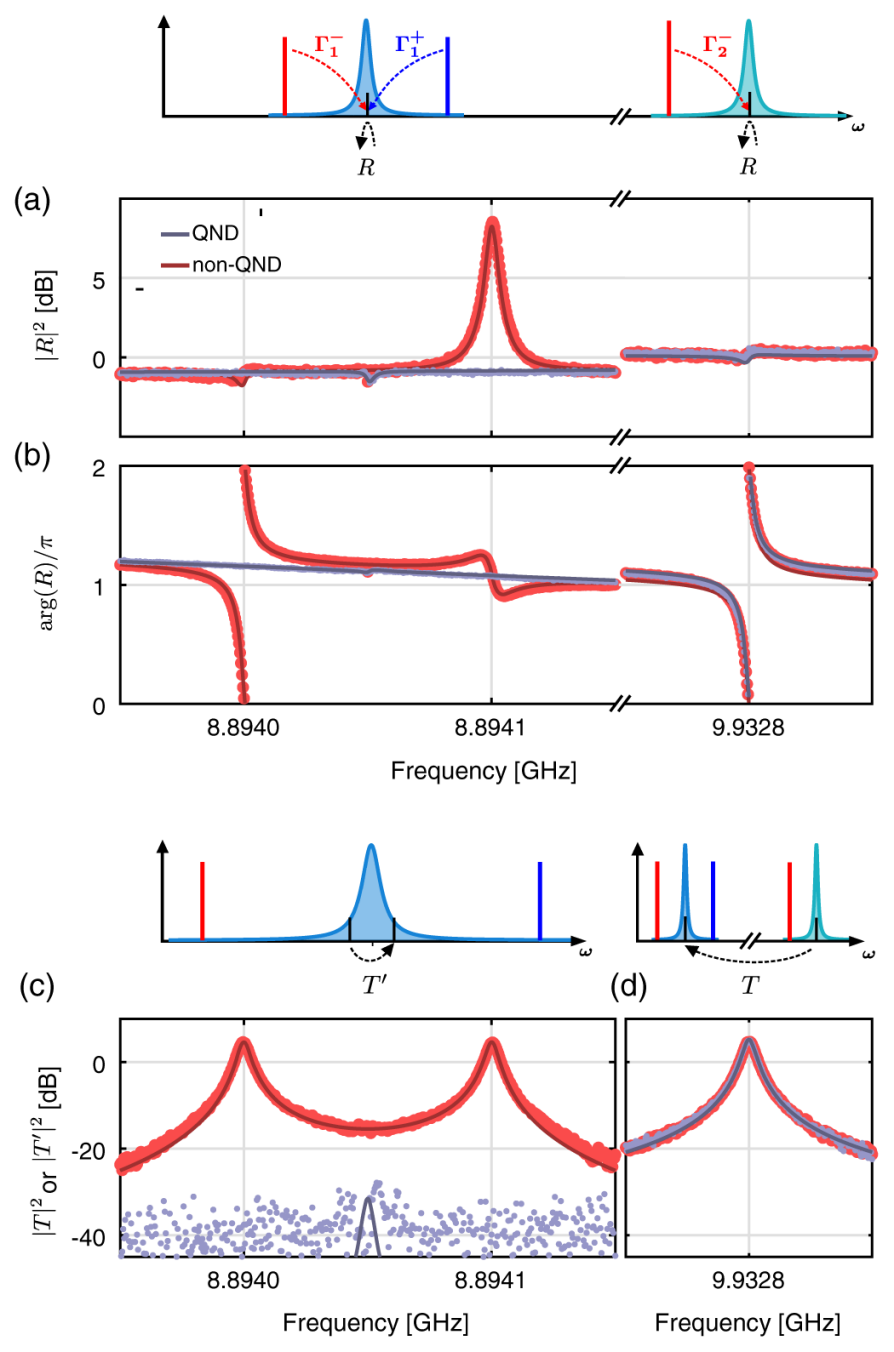
Driven responses for the QND and non-QND measurement. Data corresponding to the measurement setup in Fig. 2: the pump scattering rates are $\Gamma_{2}^{-}=2\pi \times 4.87\;\text{kHz}=529\Gamma_{m}$ and $\Gamma_{1}^{-}=\Gamma_{1}^{+}=0.9\Gamma_{2}^{-}$. (a), (b) Reflection coefficient *R* around each cavity resonance. (c) Transmission *T*′ from the upper mechanical sideband of the red measurement drive to the lower mechanical sideband of the blue measurement drive, as a function of the input frequency. A signal sent at *ω* is received at $\omega -\omega_{1}^{-}+\omega_{1}^{+}$. (d) Transmission *T* from the upper mechanical sideband of the red cooling drive to the upper mechanical sideband of the red measurement drive, as a function of the input frequency. A signal sent at *ω* is received at $\omega -\omega_{2}^{-}+\omega_{1}^{-}$. All the solid lines are from the same numerical simulation.

**FIG. 7:**
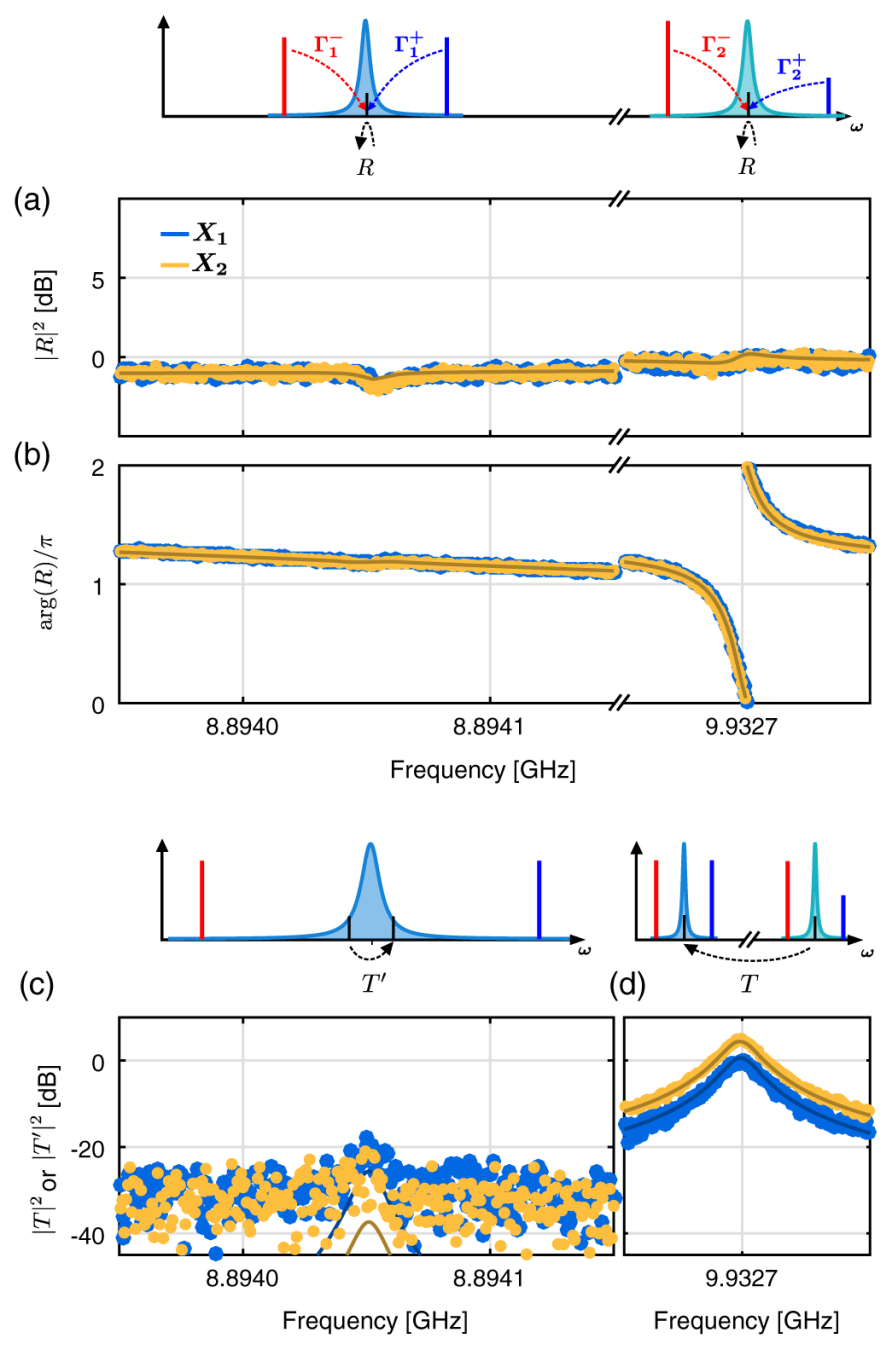
Driven responses for the tomography of the mechanical squeezed state. Data corresponding to the measurement setup in Fig. 3: the pump scattering rates are $\Gamma_{2}^{-}=2\pi \times 15.11\;\text{kHz}=1643\Gamma_{m},\Gamma_{1}^{-}/\Gamma_{2}^{-}=0.48$, and $\Gamma_{2}^{+}/\Gamma_{2}^{-}=0.07$. (a), (b) Reflection coefficient *R* around each cavity resonance. (c) Transmission *T*′ from the upper mechanical sideband of the red measurement drive to the lower mechanical sideband of the blue measurement drive, as a function of the input frequency. A signal sent at *ω* is received at $\omega -\omega_{1}^{-}+\omega_{1}^{+}$. (d) Transmission *T* from the upper mechanical sideband of the red cooling drive to the upper mechanical sideband of the red measurement drive, as a function of the input frequency. A signal sent at *ω* is received at $\omega -\omega_{2}^{-}+\omega_{1}^{-}$. All the solid lines are from the same numerical simulation.

**FIG. 8:**
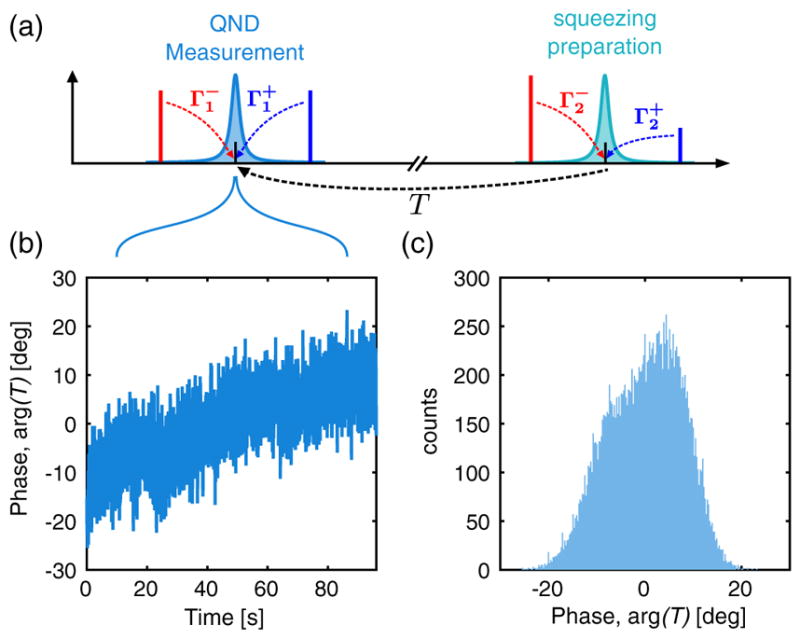
System phase stability. (a) Measurement schematic. The device is driven by four drives, at $\omega_{1,2}^{\pm }=\omega_{1,2}\pm \Omega_{m}$. A probe is applied at *ω* ≈ *ω*_2_ and received at $\omega -\omega_{2}^{-}+\omega_{1}^{-}\approx \omega_{1}$. (b) Phase of the transmission coefficient as a function of time. (c) Histogram of the time trace measured in (b).

## APPENDIX A: MECHANICAL MODE STRUCTURE

The mechanical resonator in this work is a suspended aluminum membrane fabricated following the same technique as in previous experiments [3,13,14,21,26], with two main design modifications. First, the membrane is intentionally elliptical, with a 5% difference between the major and minor diameters. The shape of the first three mechanical modes, obtained from finite element simulation, are shown in Fig. 4. The first mode is the typical drumhead mode used in previous works [3,13,26]. The ellipticity of the membrane lifts the degeneracy between the second and third modes. The geometry of the two bottom electrodes is chosen to maximize the coupling to the second mechanical mode, used in this experiment and shown in Fig. 4(b). The mechanical motion of that mode tunes the capacitance between the top electrode and each bottom electrode in an opposite way, hence imposing the sign difference between the vacuum optomechanical couplings *g*_1_ and *g*_2_. By symmetry, the third mechanical mode is decoupled from the microwave cavities.

## APPENDIX B: CALIBRATIONS AND EXPERIMENTAL DETAILS

All the drives are filtered at room temperature and attenuated in the cryostat, ensuring that they are devoid of excess noise at the cavities’ frequencies [26].

The intrinsic mechanical relaxation rate Γ*_m_* is measured from the mechanical ringdown time *τ_m_*. In Fig. 5, we show the measured ringdown time as a function of the strength of a cooling drive on the control cavity, at a frequency $\omega_{2}^{-}=\omega_{2}-\Omega_{m}$, scattering light and damping the mechanical oscillator at the rate $\Gamma_{2}^{-}$. We measure an intrinsic ringdown time *τ_m_* = 17.3 ms, corresponding to a relaxation rate Γ*_m_* = 1/*τ_m_* = 2*π* × 9.2 Hz. A comparison with theory (solid line) allows us to calibrate the cooling drive strength $\Gamma_{2}^{-}$. We calibrate in a similar manner the cooling drive strength on the measurement cavity, $\Gamma_{1}^{-}$.

The optomechanical coupling strengths for each cavity *g*_1,2_ are calibrated from a fridge temperature sweep like in Teufel *et al.* [14]. The measured values are confirmed by the measurement of the sideband asymmetry in Fig. 2.

Finally, the cavity frequencies and linewidths, the mechanical frequency, and every pump scattering rate are independently measured from the driven responses (see Appendix C).

## APPENDIX C: SCATTERING PARAMETERS

Before measuring the spectra shown in Figs. 2 and 3, we measure the driven responses of the system in the presence of all the pump drives. They contain all the information about our system, except for the bath temperatures, and represent an important calibration. With up to four drives, two cavity modes, and a mechanical mode, and with correction coming from finite sideband resolution, the analytic solutions for the driven responses can be cumbersome, if not intractable. However, they can easily be solved numerically from the linear coupled equations of motion, following methods like in Andrews *et al.* [32] or Ranzani and Aumentado [33].

In Figs. 6 and 7, we show the driven responses corresponding, respectively, to the data shown in Figs. 2 and 3. From the fit to these driven responses, we extract the cavity frequencies and linewidths, the mechanical frequency, and every pump scattering rate.

## APPENDIX D: MECHANICAL SPECTRA AND ERROR ESTIMATION

The mechanical occupancy is extracted from the total microwave power spectral density, measured at room temperature with a spectrum analyzer, following

(D1) $$\frac{{\text{PSD}}^{\pm }[\omega ]}{P_{d}}=\frac{16\eta^{2}\kappa^{2}g_{0}^{2}}{\left[\kappa^{2}{\left(1-2\eta \right)}^{2}+4\Delta^{2}][\kappa^{2}+4{\left(\Delta \pm \Omega_{m}\right)}^{2}\right]}\times \left(\frac{4n\Gamma }{\Gamma^{2}+4\delta^{2}}+\frac{4n_{\text{imp}}}{\Gamma }\right)=A\frac{S_{xx}}{x_{\text{ZP}}^{2}}+B,$$

where PSD^±^ is the measured power spectral density in [W/Hz] at the upper or lower sideband of a drive, *P_d_* is the measured drive power in [W], Δ = *ω_d_* − *ω_c_* is the drive detuning, *δ* = *ω* − *ω_d_* ± Ω*_m_* is the detuning around the mechanical sideband, *ω_c_* is the microwave cavity frequency, Ω*_m_* is the mechanical oscillator frequency, *ω_d_* is the drive frequency, Γ is the total mechanical linewidth, *κ* = *κ*_int_ + *κ*_ext_ is the total cavity linewidth, *η* = *κ*_ext_/*κ* is the coupling factor, and *g*_0_ is the optomechanical coupling strength. Here, *n*_imp_ is the measurement noise floor expressed in unit of mechanical quanta. All these parameters are extracted from the measurements described in Appendixes B and C, leaving *n* as the single unknown variable, that we extract from a Lorentzian fit of the measured noise. Its value is related to the mechanical occupancy in the following way [24,25]: for an anti-Stokes scattering process $n=n_{m}^{\text{tot}}$ (upper sideband of a single drive), for a Stokes scattering process $n=n_{m}^{\text{tot}}+1$ (lower sideband of a single drive). For the QND measurement, the mechanical sidebands of each drive interfere [7], and one obtains $n=\langle {\hat{X}}_{\Phi }^{2}\rangle =2n_{m}+1$, effectively measuring the variance of a single quadrature and avoiding the backaction.

From this measurement one can extract the apparent mechanical displacement spectral density, *S_xx_*, normalized by the variance of zero-point fluctuation, $x_{\text{ZP}}^{2}=\hbar /(2m\Omega_{m})$, where *m* is the mechanical oscillator’s mass. In Figs. 2(b) and 3(b), we show the quantity $S_{xx}/x_{\text{ZP}}^{2}\times \Gamma /4$, allowing one to read the value of *n* off of the maximum value of the Lorentzian peak. We remove the noise floor *B* for readability, as the noise floor differs slightly between the control and measurement cavities.

In Figs. 2 and 3, the error bars on the data correspond to the 90% confidence intervals, obtained from standard error propagation. The main sources of uncertainty in our experiment are due to, in order of greatest importance, the error in the Lorentzian fits of the spectra, the uncertainty in the estimation of *g*_1,2_ and, the cavities’ coupling factors *η*_1,2_.

## APPENDIX E: PHASE STABILITY OF THE DRIVES

Maintaining stable relative phases between the four microwave drives is a crucial challenge for the measurement presented in Fig. 3. Indeed, a phase variation corresponds to a rotation of the measured quadrature with respect to the squeezed quadrature. A slow phase drift occurring during the acquisition of a noise spectrum [like the one in Fig. 3(b)] averages different quadratures together, and results in a reduction of the observed squeezing.

The phase locking between the four synthesizers used here is achieved by a common 1-GHz clock. While the resulting phase stability obtained is much greater than for a typical 10-MHz clock, we still observe a drift of the relative phase between two drives of a few degrees over a minute time scale.

A more realistic measurement of the phase stability of all four drives is accessible via the driven response of the device in the presence of these drives. In Fig. 8, we show the phase of the transmission coefficient as a function of time [measured like in Fig. 7(d)]. Over a time comparable to the averaging time used for the spectrum shown in Fig. 3(b), the measured phase has varied by up to 20 deg, consistent with the reduction of the squeezing observed.

Looking forward, a better phase stability could be achieved by (1) deriving the four drives from the same local oscillator, (2) implementing an active feedback loop to lock the four drives, or (3) using a phase-coherent multichannel synthesizer.
